# Identification and Characterization of Long Non-coding RNA in Tomato Roots Under Salt Stress

**DOI:** 10.3389/fpls.2022.834027

**Published:** 2022-07-04

**Authors:** Ning Li, Zhongyu Wang, Baike Wang, Juan Wang, Ruiqiang Xu, Tao Yang, Shaoyong Huang, Huan Wang, Qinghui Yu

**Affiliations:** ^1^Institute of Horticulture Crops, Xinjiang Academy of Agricultural Sciences, Urumqi, China; ^2^Key Laboratory of Horticulture Crop Genomics and Genetic Improvement in Xinjiang, Urumqi, China; ^3^Biotechnology Research Institute, Chinese Academy of Agricultural Sciences, Beijing, China

**Keywords:** tomato, RNA-seq, lncRNA, salt stress, co-expression

## Abstract

As one of the most important vegetable crops in the world, the production of tomatoes was restricted by salt stress. Therefore, it is of great interest to analyze the salt stress tolerance genes. As the non-coding RNAs (ncRNAs) with a length of more than 200 nucleotides, long non-coding RNAs (lncRNAs) lack the ability of protein-coding, but they can play crucial roles in plant development and response to abiotic stresses by regulating gene expression. Nevertheless, there are few studies on the roles of salt-induced lncRNAs in tomatoes. Therefore, we selected wild tomato *Solanum pennellii* (*S. pennellii*) and cultivated tomato M82 to be materials. By high-throughput sequencing, 1,044 putative lncRNAs were identified here. Among them, 154 and 137 lncRNAs were differentially expressed in M82 and *S. pennellii*, respectively. Through functional analysis of target genes of differentially expressed lncRNAs (DE-lncRNAs), some genes were found to respond positively to salt stress by participating in abscisic acid (ABA) signaling pathway, brassinosteroid (BR) signaling pathway, ethylene (ETH) signaling pathway, and anti-oxidation process. We also construct a salt-induced lncRNA-mRNA co-expression network to dissect the putative mechanisms of high salt tolerance in *S. pennellii*. We analyze the function of salt-induced lncRNAs in tomato roots at the genome-wide levels for the first time. These results will contribute to understanding the molecular mechanisms of salt tolerance in tomatoes from the perspective of lncRNAs.

## Introduction

As the second-largest vegetable in the world, there is a growing demand for tomatoes. However, the area of the world’s saline-alkali land has increased annually, soil salinization has become a major impediment to affect the growth and development of tomatoes and reduce tomato production seriously (Munns, 2002). Previous studies have demonstrated that salt stress usually causes ion stress, osmotic stress, and secondary damage to plants, especially oxidative stress damage (Hamouda et al., 2016; Manishankar et al., 2018; Rady et al., 2019; Zhao et al., 2019). Therefore, in order to adapt to salt stress, plants need to rebuild the homeostasis of cell ions, osmosis, and redox balance (Yang and Guo, 2018). Research on important signaling pathways for salt tolerance have been the subject of many classic studies. So far, scientists have revealed many important signaling pathways of salt tolerance in plants. Such as the Salt Overly Sensitive (SOS) signal pathway, mitogen-activated protein kinase (MAPK) cascade signal pathway, CDPK cascade reaction pathway, ABA signal pathway, and so forth.

The transcription factor is mainly responsible for sensing and transducing early responses to salt stress in plants, such as DREB (dehydration responsive element binding factors), bZIP (basic leucine zipper), MYB (v-myb avian myeloblastosis viral oncogene homolog), and so forth. As one of the most important transcription factor families in plants, MYB transcription factors are widely involved in abiotic stress resistance, such as salt stress, drought stress and so on (Yu et al., 2017; Yao et al., 2022). Some studies have confirmed that overexpression of MYB gene can improve the salt tolerance of transgenic plants (Shen et al., 2018; Liu et al., 2020). In apple, *MdMYB46* can directly bind to the promoters of lignin biosynthesis-related genes and activate the secondary cell wall biosynthesis pathway to enhance the salt tolerance and osmotic stress ability of apple (Chen et al., 2019). In tomato, *SlMYB55* can regulate drought and salinity response by affecting the ABA-mediated signal transduction pathway, and directly or indirectly affect the expression of genes related to drought and salinity response (Chen et al., 2021b).

Salt stress can also result in an imbalance of intracellular ion homeostasis. The excessive accumulation of Na^+^ in plants will increase the Na^+^/K^+^ ratio in the cell solute, which lead to metabolic disorders (Wang et al., 2018a). Many kinds of Na^+^ transporters have been shown to maintain this homeostasis by mediating the uptake of Na^+^. For instance, *AtHKT1* can encode a plasma membrane Na^+^ transporter. Loss of function mutant of *hkt1* can inhibit salt hypersensitivity (Rus et al., 2004; Khaleda et al., 2017). In tomato and maize, the salt tolerance-related quantitative trait loci (QTL) which contained *HKT* genes had been located (Asins et al., 2013; Zhang et al., 2017). In *Solanum cheesmaniae*, gene silencing of *ScHKT1;2* could result in an increasing Na^+^/K^+^ ratio and a salt-sensitive phenotype, while the function loss of *ScHKT1;1* in rootstock could significantly reduce the ratio of Na^+^/K^+^ in leaf and flower tissues (Ra et al., 2021). Besides, *OsHKT1;5* is also a key determinant of salt tolerance in rice (Nayef et al., 2020).

In addition to these, plant hormones play key roles in cellular signal transduction and crosstalk under salt stress, such as salicylic acid (SA), jasmonic acid (JA), ethylene (ET), gibberellin (GA) and abscisic acid (ABA) (Singhal et al., 2021). SA can not only enhance the antioxidant system of plants but also promote photosynthesis under salt stress (Khan et al., 2014; Li et al., 2014b). The foliar application of SA on maize could minimize the detrimental effects of salinity (Tahjib-Ul-Arif et al., 2018). SA-mediated beneficial effects were particularly evident in the enhancement of photosynthesis-related parameters, including photosynthetic rate, carboxylation efficiency, water use efficiency, and chlorophyll content (Xue et al., 2013; Nazar et al., 2015). In *Arabidopsis thaliana* (*A. thaliana*), excessive application of SA inhibited seed germination, while an appropriate amount of SA treatment could alleviate this inhibition. As an antioxidant, melatonin can also scavenge the accumulation of ROS induced by various biotic and abiotic stresses effectively (Manchester et al., 2015). A low concentration of melatonin can improve the salt tolerance of cotton seedlings while inhibiting the expression of genes related to melatonin biosynthesis (Zhang et al., 2021). It shows that exogenous melatonin can improve the activity of the antioxidant defense system which leads to improved stress resistance in plants.

Salt stress can increase endogenous abscisic acid (ABA) levels and induce ABA-dependent and ABA-independent transcriptional regulatory networks (Roychoudhury et al., 2013). The Ca^2+^ and ABA signals can also cooperate to regulate the response to salt stress in plants (Zhu et al., 2007). Some GA-related genes, such as *AtGA2ox7* and *OsGA2ox5* have also been reported to enhance plant salt tolerance by delaying growth (Shan et al., 2014; Lange and Lange, 2015). The growth and reproduction of plants in saline-alkali soil are based on seed germination, which is closely related to ABA and GA (Yuan et al., 2011). The concentration of GA in plants changed significantly under salt stress and drought stress, indicating that GA is closely related to plant abiotic stress response (Shi et al., 2019). These results demonstrate that the molecular mechanisms resulting from salt stress tolerance are very complex in plants. Therefore, a thorough understanding of the salt tolerance mechanism is crucial.

In recent years, the role of non-coding RNA (ncRNA) in plants has attracted more and more attention. Quite a few studies have shown that ncRNA plays a crucial role in different biological processes of plants, such as cell development, regulation of epigenetics, transcription, translation, and so forth (Willmann and Poethig, 2007; Heo et al., 2013; Fonouni-Farde et al., 2021). Numerous studies show that ncRNAs have critical roles in diverse biological processes from plants to animals, such as sponging by microRNAs, cell development, acting as modular scaffolds and regulating epigenetic inheritance. The ncRNAs include rRNA, tRNA, snRNA, snoRNA, miRNA, lncRNA, and other RNAs with known functions. Long non-coding RNA (lncRNA) is a large class of transcripts from the non-coding region of the genome that contains more than 200 nucleotides but lack the protein-coding ability, and do not contain or contain a short open reading frame (ORF), usually exert a regulatory role in the response of plants to abiotic stress (Liu et al., 2015). According to the genomic positioning of its coding genes to adjacent protein-coding genes, lncRNAs can be further divided into long intergenic non-coding RNAs (lincRNAs), natural antisense transcripts (NATs), and intronic RNAs (incRNAs). With the development of technology, many lncRNAs transcripts have been identified in different plant species by tiling array and transcriptome reassembly, like *Arabidopsis*, rice, soybean and cotton, and so forth (Liu et al., 2012; Di et al., 2014; Zhang et al., 2014; Zou et al., 2016; Golicz et al., 2018; Hamid et al., 2020; Lin et al., 2020). Considering the complexity of lncRNA regulation, there are merely a few functional characteristics of lncRNAs in plants so far, but in recent years, researches on the specific role of lncRNA in plants have become more and more attractive.

Recent evidence suggests that lncRNAs play essential roles in tomatoes during flowering, resistance to *Phytophthora infestans*, fruit ripening, resistance to drought, and are also studied in *A. thaliana*, *Camellia sinensis*, and *Gossypium hirsutum* (Qin et al., 2017; Zhao et al., 2017; Deng et al., 2018; Wang et al., 2018b; Eom et al., 2019; Jiang et al., 2019b; Yang et al., 2019b; Cui et al., 2020). In recent years, competitive endogenous RNA (ceRNA) has provided an innovative way to study the molecular mechanisms of stress in plants. Among the drought stress-related lncRNAs of *Populus tomentosa*, some lncRNAs are identified as competitive endogenous RNAs, which can combine with known poplar miRNAs to regulate the expression of miRNAs target genes. At the same time, the qRT-PCR result had also verified this result (Shuai et al., 2014). Since miR398 can respond to different stresses, tae-miR398 regulates low-temperature tolerance by down-regulating its target gene *CSD1* in the cold hardiness mechanism of winter wheat. LncRNA can indirectly regulate the expression of *CSD1* by competitively binding miR398, thereby affecting the cold resistance of Dn1. The regulation of miR398 triggers a regulatory loop that is essential for cold resistance in wheat (Lu et al., 2020).

The role of lncRNAs in the process of tomato salt stress is rarely studied. However, what is not yet clear is the importance of salt-induced lncRNA in tomatoes. In comparison with cultivated tomato, wild tomato display increased salt tolerance and has stronger salt tolerance. Due to different growing environments and reproductive isolation, some salt-responsive genes in wild tomatoes may not exist in cultivated tomato species. To use modern molecular biology techniques to improve the salt tolerance of cultivated tomatoes, it is first necessary to understand the molecular mechanism of tomato salt tolerance, and wild tomatoes are important germplasm resources for revealing the salt tolerance mechanism and mining salt tolerance genes. Consequently, in this study, we selected wild tomato *Solanum pennellii* (*S. pennellii*) and cultivated tomato M82 (*Solanum lycopersicum* L.) as materials. The functions of salt-induced lncRNAs in the two cultivars are analyzed and compared by analyzing the target genes. This essay attempts to show that some salt-related genes might be regulated by salt-induced lncRNAs in *S. pennellii.* We also construct the corresponding salt-induced co-expression network. In general, this paper presents new evidence for the putative mechanism of salt tolerance in tomatoes from the perspective of the lncRNA-mRNA network.

## Materials and Methods

### Plant Materials and Stress Treatment

Seeds of cultivated tomato M82 (*S. lycopersicum*) and wild tomato (*S. pennellii*) were sown in pots containing 3:1 mixtures of vermiculite: perlite (V/V) in the growth chamber under a 16 h/8 h (day/night), and a light intensity of 100 μmol m^–2^ sec^–1^, 25°C, 20–30% relative humidity (Frary et al., 2010). Plants were cultivated for 6 weeks (well-watered and with Hoagland solution supplying at an interval of 2 weeks), and then the seedlings were exposed to 200 mM NaCl (salinity). Plants grown in the same environment without the additional stress component were used as controls. In our previous experiment, the MDA and proline levels of M82 and *S. pennellii* under salt stress had been separately determined using the MDA Assay Kit (Suzhou Comin Biotechnology Co., Ltd., Suzhou, China) and Proline Assay Kit (Suzhou Comin Biotechnology Co., Ltd., Suzhou, China) at four time points (0, 1, 12, and 24 h). From 12 to 24 h, the increase of proline content was much higher than that of 1 to 12 h. The MDA content peaked at 12 h and then began to decrease (Supplementary Figure 1). Based on this result, the roots of tomatoes were collected at 0 and 12 h following exposure to salt stress. The seeds of M82 and *S. pennellii* were collected from the Xinjiang Academy of Agricultural Sciences and TGRA (Tomato Genetic Resource Center), respectively.

### RNA Extraction, Construction of cDNA Libraries and High-Throughput Sequencing

Total RNA of tomato roots was isolated using RNAprep pure Plant Kit (Tiangen Biotech, Beijing, China) according to the manufacturer’s protocol. RNA quality and quantity were checked with NanoDrop 2000 Spectrophotometer (NanoDrop, Wilmington, DE, United States) and Agilent Bioanalyzer 2100 System (Agilent Technologies, California, CA, United States), respectively. RNA samples were pooled with equal amounts of RNA from three independent individuals. The rRNAs were removed by using the Epicentre Ribo-zero kit (Epicentre, Madison, WI, United States). Through PCR enrichment, the cDNA libraries were established. After assessing the quality of libraries, deep sequencing was performed by utilizing the Illumina sequencing platform (Illumina, San Diego, CA, United States).

### RNA-Seq Reads Mapping and Transcriptome Assembling

FastQC was utilized to check the quality of RNA-seq data. And then the adapters of raw reads that capture at least one of the following two characteristics: more than 20% of bases with a *Q*-value ≤ 20 or an ambiguous sequence content (“N”) exceeding 5% were removed. After aligning the clean reads to the reference genome by using HISAT2 with default settings (Kim et al., 2019), the reads were assembled by using Cufflinks.

### Identification of Salt-Responsive LncRNAs

To identify putative lncRNA transcripts, all the mRNA transcripts were filtered out firstly. Then the transcripts with a length of less than 200 nt or only have one exon were also filtered out. Then, the protein-coding ability of the remaining transcripts was predicted by using the CPC, PLEK, and CNCI software (Kong et al., 2007; Sun et al., 2013; Li et al., 2014a). The Swiss-Prot database was also used to filter the transcripts with any known domains (Bairoch and Apweiler, 2000). The transcripts without protein-coding ability were subsequently employed in the remainder of the study. LincRNAs, antisense lncRNAs, intronic lncRNAs, and exonic lncRNAs were classified by the cuffcompare software.

### Analysis of Differentially Expressed LncRNAs

The expression levels of lncRNAs were normalized by Transcripts Per Million (TPM). Then the R package DESeq2 was utilized to perform the differentially expressed analysis (Love et al., 2014). The fold changes of lncRNAs were calculated *via* log_2_(TPM). The lncRNAs exhibiting a | fold change| ≥ 2 and *P*-value < 0.05 were considered the DE-lncRNAs.

### Prediction of Differentially Expressed-LncRNAs Target Genes and Analysis of LncRNAs Function

The functional annotation of DE-lncRNAs was carried out by co-location and co-expression analysis. The co-locating and co-expressing lncRNA-mRNA pairs were identified by custom Perl scripts. The coding genes 1000k upstream and downstream of lncRNAs were considered to be co-located.

### GO and KEGG Enrichment Analysis

The functions of the target genes of DE-lncRNAs were annotated by GO and KEGG enrichment analysis. The GO enrichment analysis was performed on the agrigo website^1^. The KEGG analysis was performed on the KOBAS website^2^.

### Interaction Analyses of LncRNA-miRNA and miRNA-mRNA Pairs

A total of 147 known miRNA sequences of tomato were downloaded from the miRBase database^3^ and were utilized for analyzing the interaction relationship between lncRNAs and miRNAs by using the website tool psRNATarget^4^ with the default parameters (Griffiths-Jones et al., 2007; Dai et al., 2018). The interaction relationships between miRNAs and mRNAs were also implemented by the methods above for description and parameters. Finally, according to the ceRNA regulatory mechanism and the relationships between lncRNA-miRNA and miRNA-mRNA pairs, the ceRNA regulatory networks were constructed and visualized by Cytoscape software (v3.7.2) (Shannon et al., 2003).

### Validation of Target Genes of LncRNAs by qRT-PCR

To confirm the reliability of the high-throughput sequencing data, nine target genes (*ABCG36*, *ABCG37*, *ABCG40*, *CIPK5*, *CIPK6*, *CIPK10*, *SAG12*, *GSTU7*, and *GSTU8*) of salt-responsive lncRNAs were randomly selected to validate by quantitative real-time PCR (qRT-PCR). Three logical replicates were performed for each gene. Primers were designed by using Primer5 software. The relative expression levels of each gene were calculated by using the 2^–ΔΔ^ *^CT^* method (Livak and Schmittgen, 2001).

## Results

### Genome-Wide Identification and Characterization of LncRNAs in Tomatoes

**FIGURE 1:**
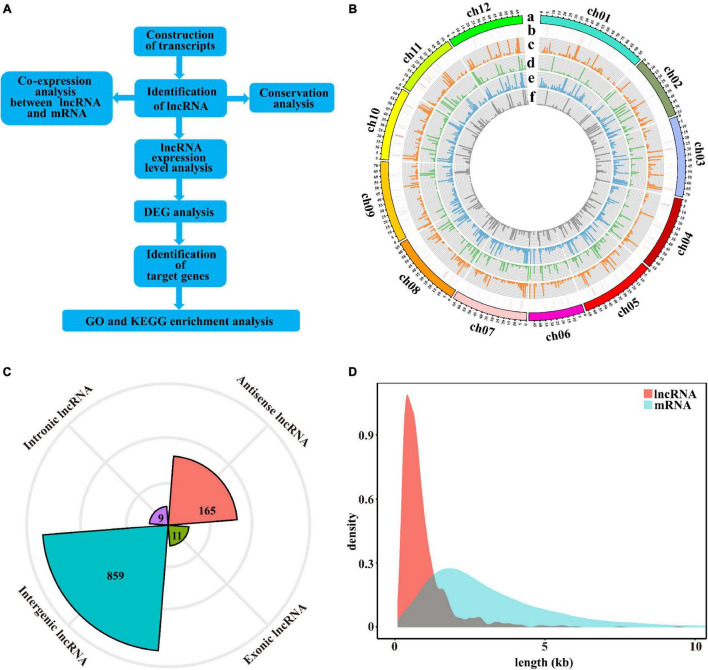
Identification and characterization of lncRNAs in tomatoes. **(A)** The pipeline for the identification of lncRNAs in tomatoes. **(B)** Circos plot depicting the distribution and expression of identified lncRNAs. From outer to inner circles, a–f circles represent chromosomes, lncRNAs distribution on chromosomes, the expression levels of lncRNAs in the control sample of M82, the expression levels of lncRNAs in the control sample of *Solanum pennellii* (*S. pennellii*), the expression levels of lncRNAs in the salt-treated sample of M82, the expression levels of lncRNAs in the salt-treated sample of *S. pennellii*, respectively. The vertical lines represent the high-low values of lncRNAs. **(C)** Radar chart represents the numbers of the four lncRNA types. **(D)** Length distribution of lncRNAs and mRNAs, orange represents lncRNAs, blue represents mRNAs.

To identify the lncRNAs in cultivated tomato and wild tomato in response to salt stress, systematically. In this study, we used roots of wild tomato and cultivated tomato under salt stress for 12 h to be materials, and then performed the whole transcriptome sequencing analysis. Each sample has three replicates. The prepared library was sequenced on the Illumina-Hiseq platform, and a total of 588.98 G reads were obtained. By removing reads containing adapter, reads containing ploy-N and low-quality reads from raw data, clean reads were eventually obtained (Supplementary Table 1). According to the analysis procedure in Figure 1A, we identified 1,044 putative lncRNAs, which were distributed on all chromosomes of tomato. Among them, the number of lncRNAs on chromosome 1 had the most lncRNAs (117 lncRNAs) and the number of lncRNAs on chromosomes 2 and 6 were the least (only 58 lncRNAs) (Figure 1B). As shown in Figure 1B, the density distribution of lncRNAs predicted in tomatoes was almost uniform with little difference. LncRNAs had the highest density on chromosome 7 (∼1.5 lncRNAs/Mbp nucleotides) and the lowest density on chromosome 3 (∼0.91 lncRNAs/Mbp nucleotides). The 1,044 lncRNAs include 859 lincRNAs, 165 antisense lncRNAs, 11 exonic, and 9 intronic lncRNAs (Figure 1C). Through further analysis of the length of these lncRNAs, it was found that the majority of them were shorter than 2,000 nt (Figure 1D) and the mean length of lncRNAs was shorter than that of mRNAs as a whole.

### Identification of Salt-Responsive Differentially Expressed-LncRNAs

**FIGURE 2:**
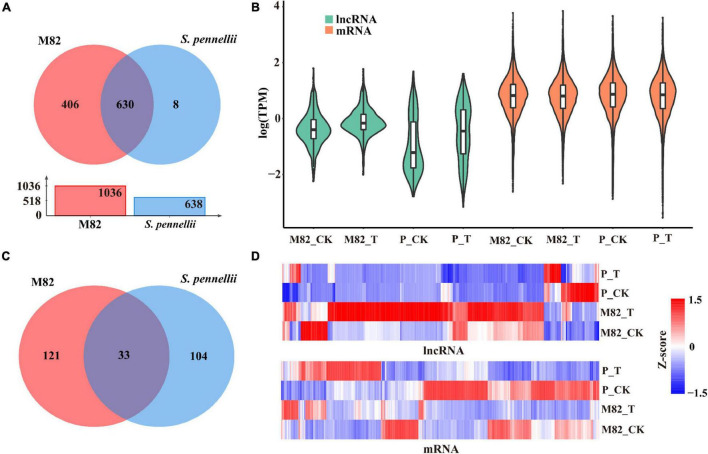
Expression patterns analysis of lncRNAs and mRNAs. **(A)** Venn diagram of common and specific lncRNAs. Red represents M82, blue represents *Solanum pennellii* (*S. pennellii*). The overlap represents the lncRNAs that were expressed in both M82 and *S. pennellii*. The red bar and blue bar in the bar graph represent the number of lncRNAs that were expressed in M82 and *S. pennellii*, respectively. **(B)** Violin plot of expression levels for lncRNA and mRNA transcripts (showed in the relative expression level of mRNA/lncRNA presented by log-transformed). **(C)** The Venn diagram shows significant DE-lncRNAs (*P*-value < 0.05). Overlaps represent the lncRNAs that were differentially expressed in both M82 and *S. pennellii*. **(D)** Heatmap presentation of relative expression levels of differentially expressed lncRNAs and mRNAs. *Z*-score represents the regulation trends, red represents up-regulation, blue represents down-regulation. The color scale bar shows *z*-score values after *z*-score row normalization.

To compare and analyze the different lncRNAs in response to salt stress in cultivated tomato and wild tomato, we compared and analyzed the expression levels of lncRNAs in response to salt stress in the two cultivars. There were 406 and 8 lncRNAs expressed specifically in M82 and *S. pennellii*, respectively. 630 lncRNAs showed expression in both cultivars (Figure 2A and Supplementary Table 2). And the average expression levels of lncRNAs were lower than that of mRNAs (Figure 2B). Subsequently, comparing the standardized expression of mRNAs between cultivated and wild tomatoes, significant differences were also founded by clustering (Figure 2C). In M82, 154 lncRNAs were significantly differential expressed, of which 133 were up-regulated and 21 were down-regulated. In contrast, 137 lncRNAs were differentially expressed in *S. pennellii*, of which 37 were up-regulated and 100 were down-regulated (Figure 2C and Supplementary Table 2). It could be seen that the expression levels of most DE-lncRNAs were significantly down-regulated in *S. pennellii*, while the expression levels of most differentially expressed lncRNAs (DE-lncRNAs) in M82 were significantly up-regulated. Only 33 lncRNAs were differentially expressed in both the two cultivars, of which 15 lncRNAs were expressed at opposite levels (Figures 2C,D). Interestingly, the expression levels of these 15 lncRNAs were all up-regulated in M82, while their expression levels were down-regulated in *S. pennellii*, indicating that these 15 lncRNAs might be relevant to the high salt tolerance of *S. pennellii*.

### *Cis*-Regulation of LncRNA Neighboring Genes

To further obtain the possible biological function of lncRNAs in tomatoes under salt stress, we treated the genes within 1 Mbps upstream/downstream DE-lncRNAs as *cis*-regulated genes and performed GO and KEGG analysis based on these genes. The results revealed that 125 DE-lncRNAs targeted 1,227 differential expressed mRNAs (DE-mRNAs) in M82 and 111 DE-lncRNAs in *S. pennellii* target 1,268 DE-mRNAs. In the end, we obtained 1,612 and 1,535 pairs of lncRNA-mRNA genes in M82 and *S. pennellii*, respectively. Among them, 700 pairs in M82 were positively correlated with expression levels, and 912 pairs were negatively correlated with expression levels. In *S. pennellii*, 880 pairs of expression levels are positively correlated, and 655 pairs of expression levels are negatively correlated (Supplementary Table 3).

**FIGURE 3:**
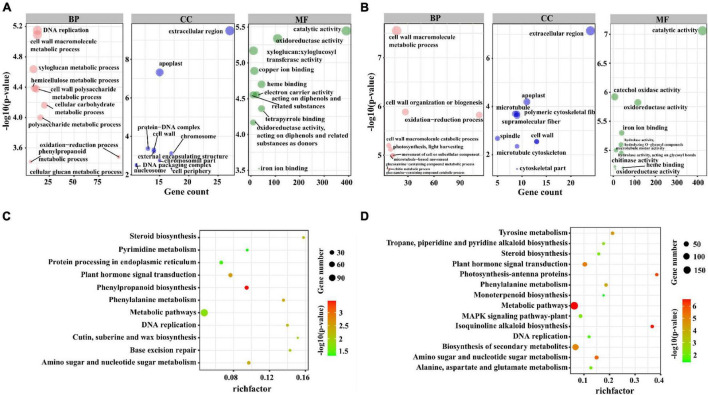
GO and KEGG enrichment analysis of the *cis*-regulated target genes of lncRNAs. **(A)** The top 10 significant terms in biological process (BP), molecular function (MF), and cellular component (CC) of GO enrichment analysis (*P*-value < 0.05) in M82. The larger circle represents a more significant enrichment of the target genes in this pathway. **(B)** The top 10 significant terms in biological process (BP), molecular function (MF), and cellular component (CC) of GO enrichment analysis (*P*-value < 0.05) in *S. pennellii*. **(C)** The KEGG enrichment scatters plot of *cis*-regulated target genes of lncRNAs in M82. The size of the circles represents the number of genes, the color of the circle represents the *P*-value. The abscissa represents the rich factor, the larger the rich factor, the greater the degree of enrichment (RichFactor is the ratio of the number of differentially expressed genes to the number of all genes in this pathway term). **(D)** The KEGG enrichment scatters plot of *cis*-regulated target genes of lncRNAs in *S. pennellii*.

In M82, the GO analysis results of these co-localized genes showed that 13, 13, 9, and 10 genes were significantly enriched in DNA replication (GO:0006260), cell wall macromolecule metabolic process (GO:0044036), xyloglucan metabolic process (GO:0010411), hemicellulose metabolic process (GO:0010410) and other terms (Figure 3A). In *S. pennellii*, the GO terms that were significantly enriched contained cell wall macromolecule metabolic process (GO:0044036), oxidation-reduction process (GO:0055114), cell wall organization or biogenesis (GO:0071554), and photosynthesis, light harvesting (GO:0009765) (Figure 3B and Supplementary Table 4). Compared with M82, there were more genes enriched in the oxidation-reduction process in *S. pennellii*, which might indicate that the steady state of redox balance in *S. pennellii* is higher than that in M82 under salt stress.

KEGG analysis results showed that these co-localized genes in M82 and *S. pennellii* were significantly enriched into 11 and 14 KEGG pathways, respectively. Among them, Phenylpropanoid biosynthesis, Base excision repair, Cutin, suberine and wax biosynthesis, Protein Processing in endoplasmic reticulum and Pyrimidine metabolism were only enriched in M82, Isoquinoline alkaloid biosynthesis, Photosynthesis-antenna proteins, Biosynthesis of secondary metabolites, Tyrosine metabolism, Tropane, piperidine and pyridine alkaloid biosynthesis, MAPK signaling pathway-plant, Alanine, aspartate and signaling pathway metabolism and Monoterpenoid biosynthesis were only enriched in *S. pennellii* (Figures 3C,D and Supplementary Table 5). Plant hormone signal transduction, Phenylalanine metabolism, Amino sugar and nucleotide sugar metabolism, DNA replication, and Steroid biosynthesis were enriched in *S. pennellii*. The number of genes enriched in these pathways was more than that of M82.

### *Trans*-Regulation of Target Genes by LncRNAs

LncRNAs could affect the expression of distant genes by regulating remote mRNA transcription. Therefore, in order to identify *trans*-regulated genes of DE-lncRNA that might be involved in the response to salt stress, we analyzed the correlation between DE-lncRNAs and DE-mRNAs (correlation coefficient *r* ≥ 0.99 or ≤−0.99). In M82, we predicted that 3,106 genes had a *trans*-regulatory relationship with 145 lncRNAs, of which 14,922 pairs are positively correlated, and 7,341 pairs are negatively correlated. In *S. pennellii*, it was predicted that 3,244 genes have a *trans*-regulatory relationship with 118 lncRNAs, of which 14,126 pairs were positively correlated, and 6,261 pairs were negatively correlated (Supplementary Table 6). These lncRNA-mRNA gene pairs were all significantly correlated (*p* < 0.05).

By comparing the expression levels of these target genes, we found that 1,685 and 1,823 genes were regulated by lncRNAs only in M82 and *S. pennellii*, respectively. There were 1,421 genes regulated by lncRNAs in both two cultivars. Among them, the expression levels of 47 genes exhibited the opposite trend, including 24 up-regulated genes in *S. pennellii* and 23 down-regulated genes in M82. For example, Solyc06g074710.1 (Hydroxyacid Hydroxycinnamoyltransferase, *HCT*), Solyc04g005810.3 (Thioredoxin-h2, *TRXH2*), Solyc08g065500.2 (Protein phosphatase 2C, *PP2C*), Solyc08g065670.3 (*PP2C*), Solyc11g010400.2 (2-oxoglutarate, *2-OG*), and Solyc11g010400.2 [Fe (II)-dependent oxygenase, *DMR6*] were significantly up-regulated in *S. pennellii* and significantly down-regulated in M82.

In M82, Solyc04g005810.3 (*TRXH2*) could be targeted by 9 lncRNAs, and their expression patterns showed a negative correlation. Two *PP2C* genes (Solyc08g065500.2 and Solyc08g065670.3) could be targeted by Lnc_000557 at the same time and exhibited the same trend in expression. Solyc11g010400.2 (*DMR6*) could be targeted by 4 lncRNAs (Lnc_000288, Lnc_000758, Lnc_000964, and Lnc_001011), of these, Lnc_000288 could also target Solyc06g074710.1 (*HCT*). In addition to the Lnc_001011-Solyc11g010400.2 pair, the other four pairs showed the same trend in expression. In *S. pennellii*, Solyc06g074710.1 (*HCT*) and Solyc11g010400.2 (*DMR6*) could be targeted by 9 lncRNAs simultaneously, and the same trend was seen between these two genes and 9 lncRNAs were the same. Beyond this, these two genes could also be targeted by two different lncRNAs (Lnc_000364-Solyc06g074710.1, Lnc_000837-Solyc06g074710.1, Lnc_000839-Solyc11g010400.2, and Lnc_000996-Solyc11g010400.2). Except for the Lnc_000837-Solyc06g074710.1 pair, the other 3 pairs showed the opposite trend in expression. In common with Solyc06g074710.1 (*HCT*), Solyc04g005810.3 (*TRXH2*) could be targeted by 3 lncRNAs (Lnc_000334, Lnc_000364, and Lnc_000600). Except for Lnc_000364-Solyc04g005810.3 (negative correlation), the expression levels of the other two pairs showed remarkable positive correlations. In contrast to M82, two *PP2C* genes (Solyc08g065500.2 and Solyc08g065670.3) could be targeted by 3 lncRNAs (Lnc_000181, Lnc_000359, and Lnc_000764) and showed remarkable positive correlations in *S. pennellii*.

**FIGURE 4:**
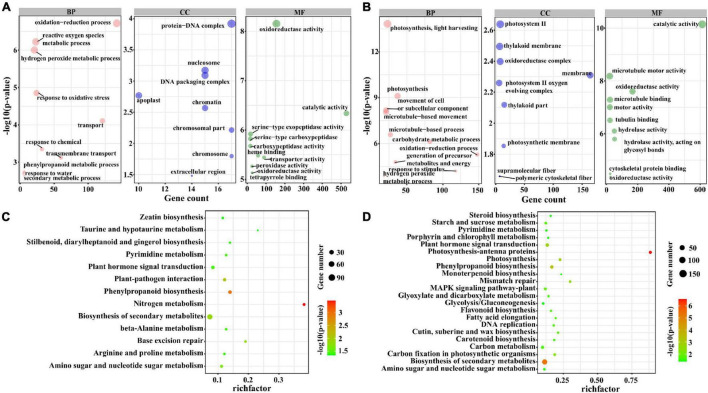
GO and KEGG enrichment analysis of the *trans*-regulated target genes of lncRNAs. **(A)** The top 10 significant terms in biological process (BP), molecular function (MF), and cellular component (CC) of GO enrichment analysis (*P*-value < 0.05) in M82. The larger the circle, the more significant of enrichment of the target genes in this pathway is. **(B)** The top 10 significant terms in biological process (BP), molecular function (MF), and cellular component (CC) of GO enrichment analysis (*P*-value < 0.05) in *S. pennellii*. **(C)** The KEGG enrichment scatters plot of *cis*-regulated target genes of lncRNAs in M82. The size of the circles represents the number of genes, the color of the circle represents the *P*-value. The abscissa represents the rich factor, the larger the rich factor, the greater the degree of enrichment (RichFactor is the ratio of the number of differentially expressed genes to the number of all genes in this pathway term). **(D)** The KEGG enrichment scatters plot of *cis*-regulated target genes of lncRNAs in *S. pennellii*.

To further investigate the potential function of lncRNAs, we divided the target genes of lncRNAs into three sets: the target genes that were regulated by lncRNAs only in M82 and *S. pennellii*, respectively, the target genes that were regulated by lncRNAs in both two cultivars. By performing the GO and KEGG enrichment analysis of these 3 sets of target genes, the differences between salt-responsive lncRNAs in two cultivars were compared. The GO enrichment results were as follows. There were 1,685 genes targeted by lncRNAs only in M82, among them, 285 genes (112 up-regulated genes and 173 down-regulated genes) were significantly enriched in 10 GO terms, including oxidation-reduction process (GO: 0055114), hydrogen peroxide metabolic process (GO: 0042743), reactive oxygen species metabolic process (GO: 0072593), response to oxidative stress (GO: 0006979), Transport (GO: 0006810), Response to Chemical (GO: 0042221), and so forth. There were 1,823 genes targeted by lncRNAs only in *S. pennellii*, among them, 381 genes (108 up-regulated genes and 273 down-regulated genes) were significantly enriched in 10 GO terms (Figures 4A,B and Supplementary Table 7), including photosynthesis, light harvesting (GO:0009765), photosynthesis (GO:0015979), movement of cell or subcellular component (GO:0006928), microtubule-based movement (GO:0007018), microtubule-based process (GO:0007017), carbohydrate metabolic process (GO:0005975), oxidation-reduction process (GO:0055114), generation of precursor metabolites and energy (GO:0006091), response to stimulus (GO:0050896), hydrogen peroxide metabolic process (GO:0042743). Interestingly, there were 3 GO terms associated with photosynthesis, which contained 37 differentially expressed genes. Except for Solyc12g017250.2 (photosystem II subunit R, *PSBR*), which was up-regulated, the other 36 genes were significantly down-regulated in *S. pennellii*. Moreover, no significant change in expression levels of the 37 photosynthesis-related genes in M82, the expression levels of these genes in M82 were also lower than that of these genes in *S. pennellii* significantly. The 37 photosynthesis-related genes could be targeted by 37 lncRNAs. There were 1,421 genes targeted by lncRNAs in both two cultivars. For these genes, cell wall biogenesis metabolic process (GO:0042546 and GO:0044036), cell wall polysaccharide metabolic process (GO:0010383), oxidation-reduction process (GO:0055114), glucan metabolic process (GO:0044042), xyloglucan metabolic process (GO:0010411) were enriched (Supplementary Figure 2).

In addition, the GO term oxidation-reduction process contained 142 and 148 genes in M82 and *S. pennellii*, respectively. In M82, 58 up-regulated and 84 down-regulated genes were significantly enriched, and 49 up-regulated and 99 down-regulated genes were enriched in *S. pennellii*. The GO term response to stimulus contained 97 and 118 genes in M82 and *S. pennellii*, respectively. Interestingly, the 97 genes in M82 were only differentially expressed in M82 and showed no significant change in *S. pennellii*. However, there were 40 genes only up-regulated differentially in *S. pennellii* among the 118 genes, which consisted of Solyc09g090970.3 (MLP-like protein 423, *MLP423*), Solyc12g056650.2 (GIGANTEA, *GI*), Solyc05g052270.2 (calcium-independent protein kinase 10, *CIPK10*), Solyc12g010130.1 (*CIPK6*), Solyc08g068960.3 (histidine kinase 5, *HK5*), Solyc02g083620.3 (ascorbate peroxidase 3, *APX3*), Solyc08g067310.1 (*CIPK5*) and some efflux protein genes. In *S. pennellii*, these 40 genes could be targeted by 27 lncRNAs with negative regulatory relationships between 10 lncRNAs and their target genes and the other 17 lncRNAs showed positive regulatory relationships with their target genes.

KEGG pathway analysis revealed that there were 197 genes (82 up-regulated genes and 115 down-regulated genes) were enriched in nitrogen metabolism, phenylpropanoid biosynthesis, plant–pathogen interaction, biosynthesis of secondary metabolites, amino sugar and nucleotide sugar metabolism, plant hormone signal transduction, and several metabolic pathways in M82. In particular, there were 17 genes enriched in the Plant hormone signal transduction pathway up-regulated significantly. In *S. pennellii*, 344 genes (105 up-regulated genes and 239 down-regulated genes) were significantly enriched in photosynthesis-antenna proteins, biosynthesis of secondary metabolites, phenylpropanoid biosynthesis, photosynthesis, amino sugar and nucleotide sugar metabolism, plant hormone signal transduction, carbon fixation in photosynthetic organisms, MAPK signaling pathway-plant and so on (Supplementary Table 8). There were 57 genes significantly enriched in photosynthesis-related pathways and most of them were down-regulated in *S. pennellii*. There were also 12 genes enriched in Plant hormone signal transduction and significantly down-regulated. Known from the comparison of KEGG results, Nitrogen metabolism, plant–pathogen interaction, base excision repair, pyrimidine metabolism, and beta-alanine metabolism were enriched only in M82 and photosynthesis-antenna proteins, photosynthesis, carbon fixation in photosynthetic organisms, and MAPK signaling pathway-plant were enriched only in *S. pennellii* (Figures 4C,D). In M82, 13 genes were enriched in the nitrogen metabolism term, of which 11 genes were down-regulated. It showed that the nitrogen metabolism pathway was severely affected. In *S. pennellii*, 22 genes were enriched in the MAPK signaling pathway-plant, of which 12 genes were up-regulated significantly. 143 genes were enriched in the biosynthesis of secondary metabolites, of which 51 genes were up-regulated significantly. In addition, phenylalanine metabolism, MAPK signaling pathway, plant hormone signal transduction, phenylpropanoid biosynthesis, and biosynthesis of secondary metabolites pathways were enriched in both two cultivars (Supplementary Figure 3). These results illustrated that the salt-responsive pathways in M82 and *S. pennellii* were significantly different, lncRNA might be involved in the response to salt stress by regulating their potential target genes. Apart from that, these lncRNAs might play an essential role in enhancing salt tolerance in *S. pennellii*.

### Construction of LncRNA-mRNA Networks

**FIGURE 5:**
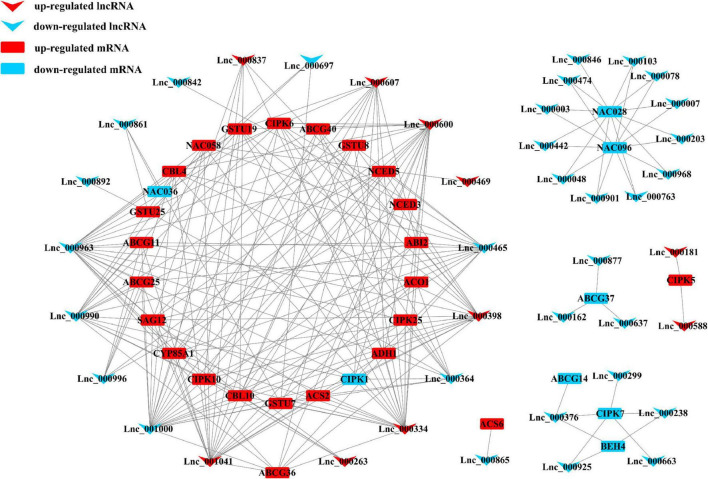
Representatives of the interaction networks among lncRNAs and target protein-coding genes in *S. pennellii*. Red down arrows refer to up-regulated lncRNAs, blue down arrows refer to up-regulated lncRNAs. Red rectangles refer to up-regulated mRNAs, blue rectangles refer to down-regulated mRNAs.

In order to analyze and compare the functions of lncRNAs and the relationship between lncRNAs and their targeted mRNAs in two tomato cultivars under salt stress, we constructed several putative networks with Cytoscape (Figure 5 and Supplementary Figure 4). As showcased in the figures, complex networks were observed. Among them, several genes were found to be involved in oxidation/reduction reaction, phytohormone signaling, and biosynthesis-related in *S. pennellii*, such as *ABI2*, *ACO1*, *CIPK5/25*, *CBL4/10*, *CYP85A1*, *ABCG25*, *NCED3/5*, and so forth. These 32 mRNAs were predicted to be targeted by 41 lncRNAs (Figure 5). In M82, 13 genes were involved in nitrogen metabolism under salt stress and regulated by 39 lncRNAs (Supplementary Figure 4). Some transcription factors like MYB were also found to participate in the process of phenotropic biosynthesis. They might activate downstream salt-responsive genes under salt-stress conditions. And 25 signal transduction-related genes were also found to be targeted by 78 lncRNAs. Most of them were up-regulated significantly. These relationships among lncRNAs and target genes might play vital roles in sensing and responding to salt stresses.

### The Function of LncRNAs Acting as miRNA Targets in Tomatoes

Previous studies have indicated that the function of lncRNAs could be achieved by targeting miRNAs. Through the psRNATarget server, we screened out the salt-responsive lncRNAs that might be targeted by miRNAs in M82 and *S. pennellii* and established the potential relationships between lncRNAs and miRNAs under salt stress.

In M82, 23 lncRNAs were predicted to be targets of 34 miRNAs from 24 miRNA families, and 21 lncRNAs were predicted to be targets of 28 miRNAs from 20 miRNA families in *S. pennellii*. There were 3 lncRNAs (Lnc_000547, Lnc_000693, and Lnc_000976) that could be targeted by miRNAs in both two cultivars. Lnc_000547 and Lnc_000693 could be targeted by sly-miR9478-3p and sly-miR403-5p, respectively, while Lnc_000976 could be targeted by 4 miRNAs (sly-miR156e-5p, sly-miR171c, sly-miR395a, and sly-miR395b). There were 20 and 18 lncRNAs that could be targeted by the miRNAs in M82 and *S. pennellii*, respectively. In M82, Lnc_000518, Lnc_000563, and Lnc_000726 were the top 3 lncRNAs that connected with miRNAs (7, 6, and 5 miRNAs could be connected, respectively). In *S. pennellii*, Lnc_000360, Lnc_000976, and Lnc_001000 were the top 3 lncRNAs that connected with miRNAs (6, 4, and 4 miRNAs could be connected, respectively).

**FIGURE 6:**
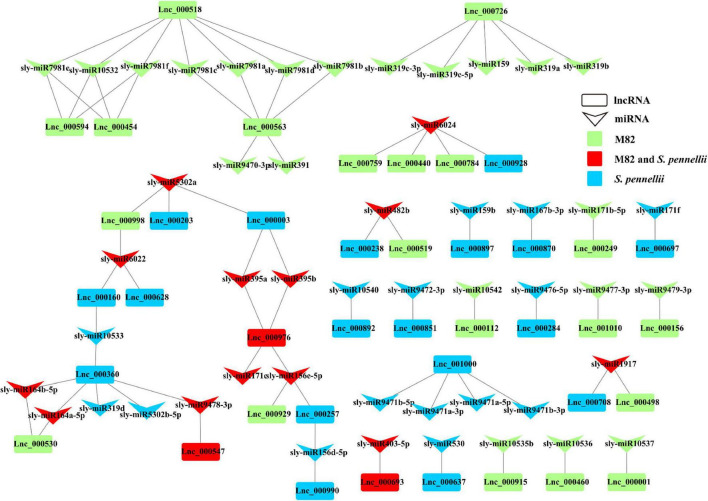
LncRNA–miRNA interaction network. Down arrows refer to miRNAs, rectangles refer to lncRNAs. Green represents the lncRNA that could be targeted by miRNAs only in M82 (or the miRNA that can target DE-lncRNA only in M82). Blue represents the lncRNA that could be targeted by miRNAs only in *S. pennellii* (or the miRNA that can target DE-lncRNAs only in *S. pennellii*). Red represents the lncRNA that could be targeted by miRNAs in both M82 and *S. pennellii* (or the miRNA that can target DE-lncRNAs in both M82 and *S. pennellii*).

Many salt-responsive miRNAs were already discovered in plants, miR156, miR159, miR164, miR167, miR171, miR319, miR395, and so forth (Sunkar and Zhu, 2004; Patade and Suprasanna, 2010; Wang et al., 2013; Qin et al., 2015; Liu et al., 2019; Hernandez et al., 2020; Ye et al., 2020; Makkar et al., 2021). In *S. pennelli*, sly-miR156d-5p, sly-miR156e-5p, sly-miR395a, sly-miR395b, sly-miR5302a and sly-miR6022 could target 2 lncRNAs. This illustrated that they could respond to salt stress by the miRNA-lncRNA interactions. Sly-miR156d-5p and sly-miR156e-5p could target Lnc_000990 and Lnc_000976, respectively. Lnc_000257 could be targeted by sly-miR156d-5p and sly-miR156e-5p. Both sly-miR395a and sly-miR395b could target Lnc_000003 and Lnc_000976. Sly-miR5302a could target 2 lncRNAs (Lnc_000003 and Lnc_000203). Sly-miR6022 could target Lnc_000160 and Lnc_000628. While in M82, Lnc_000998 could also be targeted by sly-miR5302a and sly-miR6022 (Figure 6). These pieces of evidence indicated that lncRNAs could be involved in the gene regulation process through the miRNA–lncRNA interactions under salt stress in tomatoes. The function of these lncRNAs remained to be further determined.

### Validation of Target Genes of Salt-Responsive LncRNAs

**FIGURE 7:**
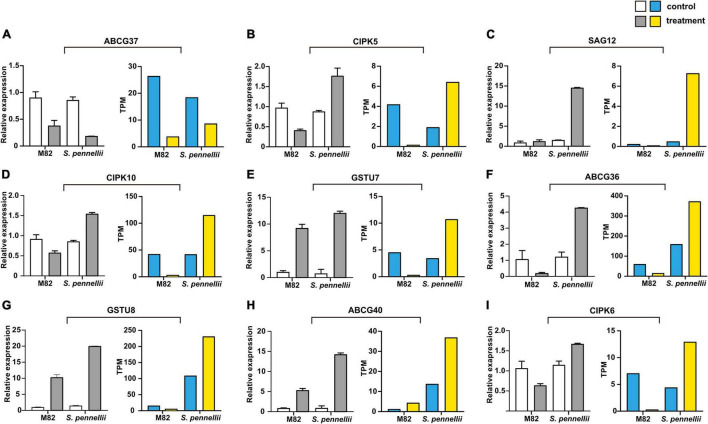
qRT-PCR validation of the target genes of salt-responsive lncRNAs. **(A–I)** The qRT-PCR results are represented on the left graphs, the RNA-seq results of mRNAs are represented on the right graphs. The error bars represent the standard error of replicates. The white and blue bars represent the control samples, the gray and yellow bars represent the salt treatment samples.

To confirm the reliability of the high-throughput sequencing data, quantitative real-time PCR (qRT-PCR) was utilized to compare expression patterns of salt-responsive genes. Nine target mRNAs of salt-responsive lncRNAs were randomly selected. Between the qRT-PCR and RNA-seq results, a similar tendency was observed, suggesting that the results of Illumina sequencing were trustable. Among these genes, all genes were significantly up-regulated in *S. pennellii* except *ABCG37*. Only *ABCG40* was up-regulated in M82. However, some discordant results were also observed between qRT-PCR and RNA-seq results in M82, like *GSTU7* and *GSTU8*. This might be due to their low level of expression in M82 (Figure 7).

## Discussion

In the past few years, the role of lncRNAs during the normal development process and under abiotic stress in tomatoes has been identified. For instance, the flowering, fruit cracking, response to drought, disease resistance, and CRISPR/cas9 technology verified that lncRNA1459 was indeed involved in tomato fruit ripening (Li et al., 2018; Eom et al., 2019; Jiang et al., 2019b; Yang et al., 2019b; Xue et al., 2020). With respect to stress-related lncRNAs in other plants, which have been reported according to previous studies. The new lncRNA MuLnc1 in *Morus multicaulis* could regulate mul-miR3954 to produce si161579, which in turn inhibited the expression of *MuCML27*. The overexpression of *MuCML27* could improve the salt stress tolerance of plants (Gai et al., 2018). The 1,117 salt-responsive lncRNAs in *Gossypium hirsutum* were also reported. In cotton, transgenic plants that increased in seed germination rate, fresh weight, and root length could be obtained by overexpression of lncRNA973, while knockout of lncRNA973 could obtain the plants with more severe wilting and leaf abscission symptoms (Zhang et al., 2019). There were 126 and 133 DE-lncRNAs were also identified in salt-tolerant and salt-sensitive varieties of *Sweet Sorghum*, respectively (Sun et al., 2020). These lncRNAs might play a role as ceRNAs to affect the response to salt stress by regulating the expression of target genes related to ion transport, protein modification, and transcriptional regulation in plants. To the best of our knowledge, this is the first report of the function of lncRNAs in response to salt stress in wild and cultivated tomatoes. The phenotypes of tomatoes under salt stress, such as developmentally arrested seedlings and restricted root growth and development were shown (Kesten et al., 2019). Therefore, it is important to understand the molecular mechanisms underlying the adaptation of tomatoes to salt stress. As a more salt-tolerant tomato genotype, *S. pennellii* showed relatively more salt-responsive compared to cultivated tomato M82. Identifying the unique genes that are involved in salt tolerance in wild tomatoes is essential for cultivating new salt-tolerant tomato cultivars.

In this study, we identified 1,044 unique lncRNAs in two cultivars according to strict standards. Some lncRNAs might be excluded during the selection process, but these 1,044 lncRNAs could be considered as a group of reliable tomato lncRNAs. Based upon the genome location, these lncRNAs could be divided into four types, including intergenic lncRNAs, antisense lncRNAs, exonic and intronic lncRNAs. We also identified 154 and 137 lncRNAs that were differentially expressed in M82 and *S. pennellii*, respectively. Interestingly, about 86% DE-lncRNAs in M82 were significantly up-regulated, while about 73% DE-lncRNAs in *S. pennellii* were significantly down-regulated. This result revealed a significant difference in the responses of these salt-responsive lncRNAs between cultivated and wild tomatoes under salt stress conditions.

Previous studies showed that lncRNAs had a less conserved sequence, which suggested that the DE-lncRNAs in *S. pennellii* might be associated with its high salt tolerance, so the functional elucidation of these DE-lncRNAs mattered deeply (Liu et al., 2018). For the most part, lncRNAs can regulate the expression of neighboring genes by *cis*-regulation and genes located on different chromosomes by *trans*-regulation (Rossetto et al., 2013). lncRNA can also regulate mRNA expression mediated by miRNAs (Zhou et al., 2019). Since the salt-tolerance mechanism of *S. pennellii* has not been systematically studied so far. However, lncRNAs have been identified to be an important regulator in response to salt stress in recent years. Therefore, we performed comparative analyses of the salt-responsive lncRNAs target genes between M82 and *S. pennellii*. According to the results, we found that some target genes have been confirmed to be related to the salt-tolerance process. In spinach, overexpression of the brassinosteroid (BR) biosynthetic gene *CYP85A1* could enhance salt tolerance (Duan et al., 2017). Solyc02g089160.3 (*CYP85A1*) was found to be significantly induced in both two cultivars, indicating that the BR content might be related to salt tolerance in tomatoes (Ashraf et al., 2010; Rattan et al., 2020). As one of the key enzymes from the ethylene (ETH) biosynthetic pathway, *ACO1* was significantly upregulated in M82 and could be regulated by five lncRNAs (Lnc_000518, Lnc_000635, Lnc_000693, Lnc_000750, and Lnc_001010). This suggested that these five lncRNAs might play a role in the ethylene synthesis pathway by targeting *ACO1* under salt stress. The *ACO1* was also involved in the ethylene signal transduction process and could be induced under other stresses (Han et al., 2010; Jia et al., 2013).

The SALT OVERLY SENSITIVE 3 (*SOS3*/*CBL4*) and SOS3-LIKE CALCIUM BINDING PROTEIN8 (*SCABP8*/*CBL10*) help plants cope with Na^+^ toxicity through mediating Ca^2+^ signaling in roots and shoots, respectively (Quan et al., 2007). In this study, Solyc08g065330.3 (calcineurin B-like protein 10, *CBL10*), which was *trans*-regulated by 11 and 13 lncRNAs in M82 and *S. pennellii*, respectively, was significantly up-regulated in both two cultivars. This indicated that *CBL10* could be induced ubiquitously in tomatoes under salt stress to help plant scope with Na^+^ toxicity. *ADH1* was confirmed to play an important role in stress response in plants (Shi et al., 2017). Under salt stress, *ADH1* is significantly induced and *ADH1* overexpressing plants showed improved salt stress resistance in Arabidopsis. In this study, *ADH1* was significantly up-regulated and could be regulated by 9 and 12 lncRNAs in M82 and *S. pennellii*, respectively.

The plant phytohormones have been implicated as important regulators of plant response to abiotic stress, like BR, jasmonic acid (JA), gibberellin (GA), and ethylene (ETH), etc. (Magome et al., 2008; Jiang et al., 2013; Garcia-Abellan et al., 2015; Wei et al., 2015; Zhu et al., 2016). We found some genes that might be plant hormone biosynthesis and signal transduction component-encoding genes that were significantly induced in *S. pennellii*. ABA signaling plays an essential role in response to external abiotic stresses. Whereas *NCED3*, the key gene for ABA synthesis, is considered to be important for the emergence of ABA signals (Takahashi et al., 2018). It can also be regulated by multiple genes, for instance, the ATAF1 transcription factor, NGTHA1, and HDA15 (Jensen et al., 2013; Sato et al., 2018; Truong et al., 2021). The expression of *NCED3* was significantly induced in *S. pennellii*. As a target gene, Lnc_000842 and Lnc_000996 might be involved in the ABA signaling pathway under salt stress through the transaction on *NCED3*. In rice, *NCED5* was shown to be induced by salt stress. The *nced5* mutant had reduced ABA levels, the ability of the mutant plants to tolerate salt and water stress was also impaired. Furthermore, overexpression of *NCED5* could increase ABA levels and enhance tolerability (Huang et al., 2019). The expression of *NCED5* was significantly induced and predicted to be targeted by 10 lncRNAs. This indicated that lncRNAs might affect ABA levels in *S. pennellii* by regulating the expression levels of *NCED3* and *NCED5* (Barrero et al., 2006). *AtABCG25* can participate in the intercellular ABA signaling pathway as an ABA transmembrane transporter in *A. thaliana* (Kuromori et al., 2010), and overexpression of *AtABCG25* can enhance the ABA accumulation in guard cells and improve plant water use efficiency (Kuromori et al., 2016). Solyc11g018680.1 (*AtABCG25*) could be significantly induced in *S. pennellii* and targeted by 6 lncRNAs. Besides, *AtABCG25* can also transport ABA together with *AtABCG40* to achieve stomatal regulation (Kuromori and Shinozaki, 2010; Kang et al., 2015). Solyc09g091670.3 (*AtABCG40*) was predicted to be targeted by 6 and 7 lncRNAs in M82 and *S. pennellii*, respectively. It was significantly up-regulated in two cultivars. In contrast to M82, lncRNAs might enhance ABA signaling transduction by targeting *AtABCG25* and *AtABCG40* simultaneously to adapt to salt stress *via* more rapid stomatal regulation. As a synergid-expressed kinase, FERONIA plays a critical role in hormone signaling and stress tolerance in different plants. While the FERONIA activity could be regulated by *ABI2* (Feng et al., 2018). In *S. pennellii*, *ABI2* was significantly induced. 10 lncRNAs might mediate FERONIA signaling pathway by *cis*-regulating *ABI2* expression. Ethylene is an important plant hormone involved in plant growth, development and response to environmental stresses (Johnson and Ecker, 1998). 1-aminocyclopropane-1-carboxylic acid synthase (ACS) is a key rate-limiting enzyme responsible for ethylene biosynthesis in plants (Yang and Hoffman, 1984). And the ethylene synthesis pathway is tightly regulated by exogenous and endogenous signals at the transcriptional and post-transcriptional levels. MAPK phosphorylation-induced stabilization of ACS6 protein is mediated by the non-catalytic C-terminal domain, which also contains the *cis*-determinant for rapid degradation by the 26S proteasome pathway. In *Arabidopsis*, as the MAPK substrates, *ACS2* and *ACS6* could be phosphorylated by *MPK6* and results in ACS accumulation and induction of ethylene (Xu et al., 2008; Tsuda et al., 2013). *ACS6* was found to be induced by Lnc_000865 only in *S. pennellii*, while *ACS2* could be induced in both two cultivars. It showed that *ACS2* might be necessary under salt stress to initiate ethylene biosynthesis in tomatoes, while the up-regulated *ACS6* might enhance the biosynthesis of ethylene to improve the salt tolerance of *S. pennellii*. The previous study had shown that *DWF4*, as a BR biosynthesis gene, could be directly inhibited by *BZR1* and that its expression level increased at an early stage of stress (He et al., 2005; Kim et al., 2013). A homolog of *BZR1* called *BEH4* was found to be targeted by 3 lncRNA and inhibited in *S. pennellii*. As the target gene of *BZR1*, *DWF4* was significantly up-regulated. It showed that lncRNAs might mediate the BR biosynthesis process to enhance the salt tolerance in *S. pennellii*.

Previous studies have shown that high [Ca^2+^] cyt can be sensed by (CBLs). However, CBL4 and CBL10, a SOS3-like calcium-binding protein, can help plants cope with Na^+^ toxicity by mediating Ca^2+^ signals in roots and shoots, respectively. Acting as the main Ca^2+^ sensor, CBLs can bind Ca^2+^ and regulate the activity of many proteins (Quan et al., 2007; Yang et al., 2019a; Chai et al., 2020). Solyc12g055920.2 (*CBL4*) could be induced in *S. pennellii* and no significant changes were noted in M82. In *Arabidopsis*, *CBL4* can interact with *CIPK6* to regulate the activity of the K^+^ channel protein (AKT2) (Held et al., 2011). *CBL4* was also shown to interact with *CIPK5* in bermudagrass, and overexpression of either *CBL4* or *CIPK5* could increase salt tolerance (Huang et al., 2020). Solyc08g067310.1 (*CIPK5*) could be targeted by 2 lncRNAs (Lnc_000181 and Lnc_000588) induced in *S. pennellii*, while Solyc12g010130.1 (*CIPK6*) could be targeted by 9 lncRNAs and induced only in *S. pennellii*. It showed that lncRNAs in *S. pennellii* might help plants cope with Na^+^ toxicity by regulating Ca^2+^ signaling in the root rapidly. Tau class glutathione transferases (GSTU) genes could protect plants from oxidative injury, overexpression of SbGST showed higher germination rates (Jha et al., 2011; Tiwari et al., 2016). In this study, Solyc06g069045.1 (*GSTU7*) and Solyc09g011510.2 (*GSTU8*) were both induced in *S. pennellii*. Previous studies showed that NAC proteins were involved in plant salt stress response (Jeong et al., 2013; Jiang et al., 2019a). In *Reaumuria trigyna*, *NAC100* could interact with *RtRbohE*/*SAG12* to accelerate salt-induced programmed cell death. *NAC100* promoted reactive oxygen species, Ca^2+^, and Na^+^ accumulation and increased the Na^+^/K^+^ ratio (Ma et al., 2021a). Interestingly, both *NAC100* and *SAG12* were differentially up-regulated in *S. pennellii* and 10 lncRNAs could target these two genes. Furthermore, Lnc_000364 and Lnc_000263 could target *NAC100* and *SAG12*, respectively. It illustrated that *S. pennellii* might adapt to the salt stress environment by regulating the programmed death process of cells *via* lncRNAs.

Moreover, one of the molecular mechanisms by which lncRNAs regulate the expression of genes is to interact with miRNAs as ceRNAs. By using psRNAtarget, 41 lncRNAs were predicted as potential targets of 49 miRNAs. In particular, miR156 was confirmed to be associated with salt stress, and overexpression of miR156a could reduce the salt tolerance of apples (Ma et al., 2021b). In maize, zma-miR159 was up-regulated under salt stress, while mir159 could affect the ABA signaling regulatory process by interacting with its target gene *MYB*. Whereas zma-miR164 could respond to the salt stress by targeting the NAC transcription factors (Shan et al., 2020). In *Populus euphratica*, peu-miR164 was predicted to target *PeNAC070*. Overexpression of *NAC070* could reduce salt tolerance in *Arabidopsis* (Lu et al., 2017). It indicated that miR164 is a positive regulator of the salt tolerance pathway in Populus euphratica. MiR319 plays important role in response to abiotic stress in some C3 plants, like *Arabidopsis* and rice, for example. In *Panicum virgatum* L., where researchers found that miR319 could modulate the salt response of switchgrass by fine-tuning ET synthesis. Different lncRNAs were specifically targeted by these salt-related miRNAs in both two cultivars. In *S. pennellii*, Lnc_000257 and Lnc_000990 could be targeted by sly-mir156d-5p, Lnc_000257 and Lnc_000976 could be targeted by sly-miR156e-5p. Lnc_000360 could be targeted by 3 miRNAs (sly-miR164a-5p, sly-miR164b-5p, and sly-miR319d). While in M82, Lnc_000929 and Lnc_000976 could be targeted by sly-miR156e-5p, sly-miR164a-5p, and sly-miR164b-5p could target Lnc_000530. These results suggested that these lncRNAs might respond to the salt stress process by interacting with miRNAs and had significant differences between the two tomato cultivars.

In summary, we found that some pathways such as phytohormone metabolism, photosynthesis, and protein/amino acid metabolism were closely related to salt stress by analyzing the function of lncRNA target genes in M82 and *S. pennellii*. Phytohormones regulate many growths, developmental processes, and responses to various stresses. Following the target genes prediction, we obtained some lncRNAs that were involved in phytohormone biosynthesis and transport pathways. Under salt stress, the expression levels of some genes involved in hormone synthesis including ABA and ethylene were significantly up-regulated in *S. pennellii*, and the mean expression levels were also higher than that in M82, which might account for the differences in salt tolerance of cultivated and wild genotypes. Increased cell death-related gene expression in *S. pennellii* might result in having the chance to receive more death signals to induce apoptosis and contribute to the tolerance to salt stress. In addition, we also analyzed the relationships between salt-responsive lncRNAs and miRNAs to elucidate the roles and distinction of salt-responsive lncRNAs in wild and cultivated tomatoes from several different standpoints. We also constructed several putative salt tolerance-related networks that were associated with the high salt tolerance of *S. pennellii*. In particular, we will expect to analyze the functions of these salt-responsive lncRNAs by utilizing more scientifically rigorous methods.

## Data Availability Statement

The datasets presented in this study can be found in online repositories. The names of the repository/repositories and accession number(s) can be found below: https://ngdc.cncb.ac.cn/gsub/submit/gsa/list/, CRA004289.

## Author Contributions

QY, HW, and NL conceived and designed the experiments. BW and JW performed the experiments. NL, RX, TY, and SH gathered samples. QY supported the materials. ZW participated in the analysis of the data and drafted the manuscript. NL and ZW revised the manuscript. All authors have read and approved the final version of the manuscript.

## Conflict of Interest

The authors declare that the research was conducted in the absence of any commercial or financial relationships that could be construed as a potential conflict of interest.

## Publisher’s Note

All claims expressed in this article are solely those of the authors and do not necessarily represent those of their affiliated organizations, or those of the publisher, the editors and the reviewers. Any product that may be evaluated in this article, or claim that may be made by its manufacturer, is not guaranteed or endorsed by the publisher.

## Footnotes

1
http://systemsbiology.cau.edu.cn/agriGOv2/

2
http://kobas.cbi.pku.edu.cn/kobas3

3
https://www.mirbase.org

4
http://plantgrn.noble.org/psRNATarget/

## Supplementary Material

Supplementary Figure 1 The MDA and proline level of M82 and *S. pennellii* at 0, 1, 12, and 24 h under salt stress.

Supplementary Figure 2 GO enrichment results of *trans*-regulated target genes pairs in both M82 and *S. pennellii*.

Supplementary Figure 3 KEGG enrichment results of *trans*-regulated target genes pairs in both M82 and *S. pennellii*.

Supplementary Figure 4 The relationships between DE-lncRNAs and DE-mRNAs in M82. Down arrows refer to lncRNAs, rectangles refer to mRNAs. Red represents the up-regulated lncRNA or mRNA. Blue represents the down-regulated lncRNA or mRNA. The lines between lncRNAs and mRNAs represent the targeting relationships.

Supplementary Table 1 RNA-seq data quality assessment.

Supplementary Table 2 Differentially expressed lncRNAs in M82 and *Solanum Pennellii*.

Supplementary Table 3 *Cis*-regulated lncRNA-mRNA pairs in M82 and *S. pennellii*.

Supplementary Table 4 GO enrichment results of *cis*-regulated target genes pairs in M82 and *S. pennellii*.

Supplementary Table 5 KEGG enrichment results of *cis*-regulated target genes pairs in M82 and *S. pennellii*.

Supplementary Table 6 *Trans*-regulated lncRNA-mRNA pairs in M82 and *S. pennellii*.

Supplementary Table 7 GO enrichment results of *trans*-regulated target genes pairs in M82 and *S. pennellii*.

Supplementary Table 8 KEGG enrichment results of *trans*-regulated target genes pairs in M82 and *S. pennellii*.
