# Supplementary figures and images for: Identification and Characterization of Long Non-coding RNA in Tomato Roots Under Salt Stress
Source: Front Plant Sci. 2022 Jul 4;13:834027. doi: 10.3389/fpls.2022.834027 (PMC9295719; doi:10.3389/fpls.2022.834027)

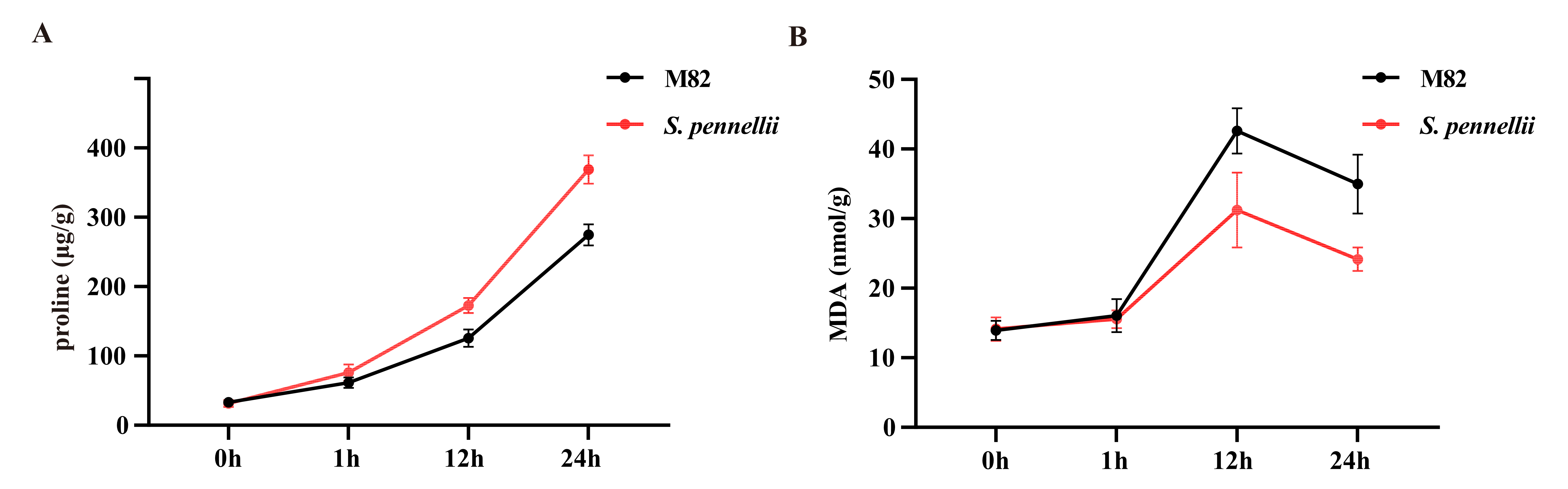

Supplement: Supplementary Figure 1 — The MDA and proline level of M82 and S. pennellii at 0, 1, 12, and 24 h under salt stress. [file Image_1.TIF]

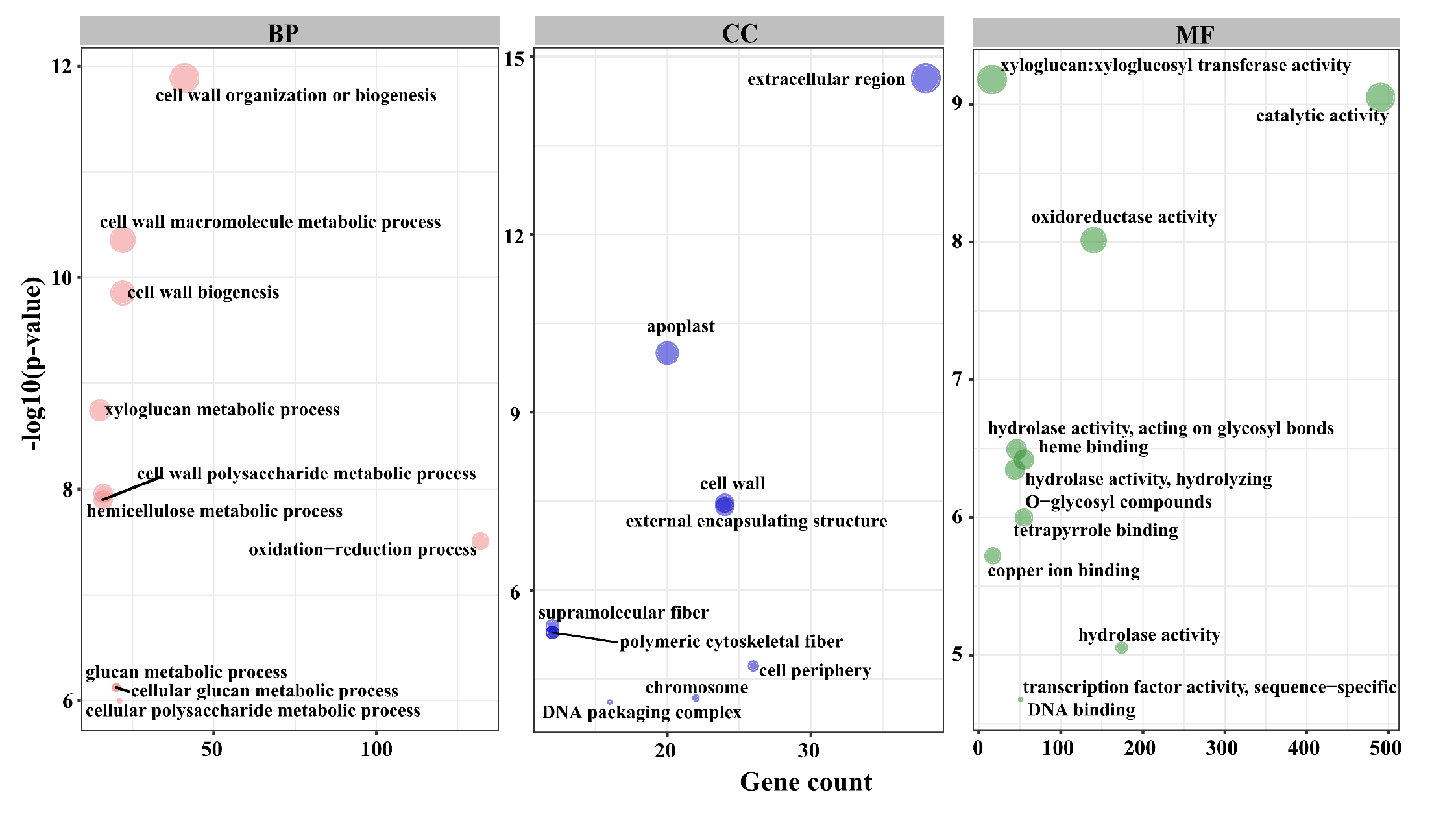

Supplement: Supplementary Figure 2 — GO enrichment results of trans-regulated target genes pairs in both M82 and S. pennellii. [file Image_2.TIF]

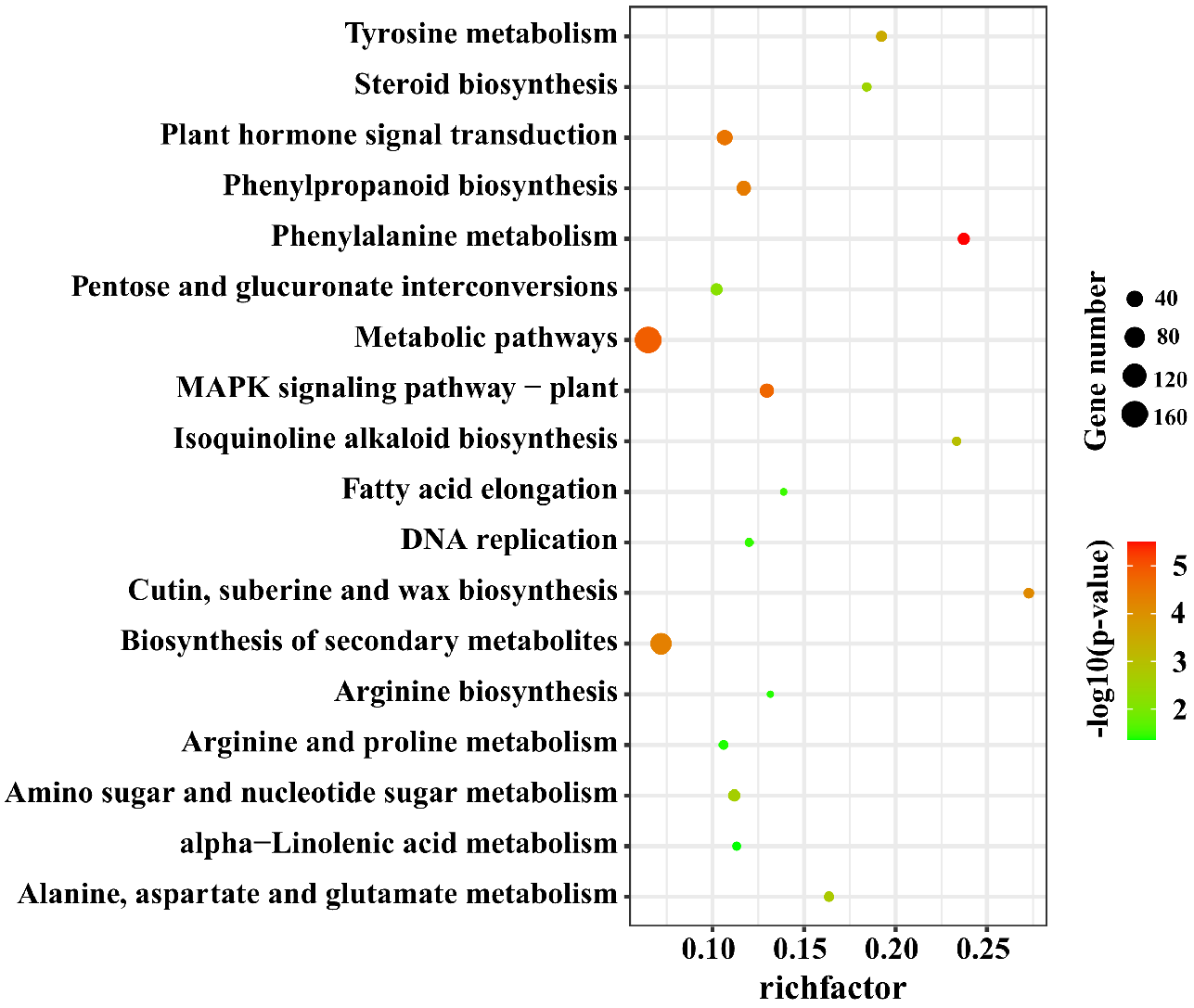

Supplement: Supplementary Figure 3 — KEGG enrichment results of trans-regulated target genes pairs in both M82 and S. pennellii. [file Image_3.TIF]

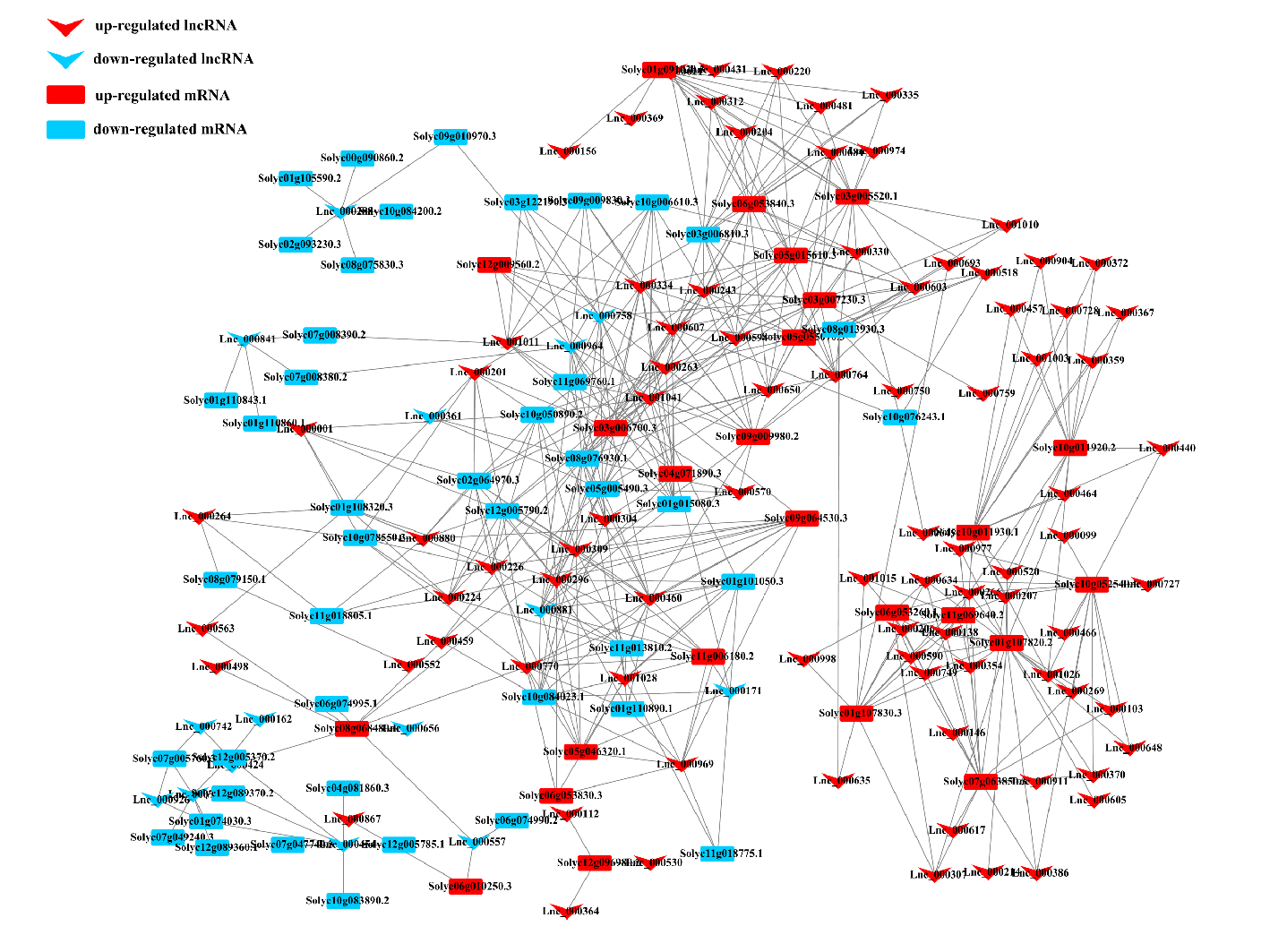

Supplement: Supplementary Figure 4 — The relationships between DE-lncRNAs and DE-mRNAs in M82. Down arrows refer to lncRNAs, rectangles refer to mRNAs. Red represents the up-regulated lncRNA or mRNA. Blue represents the down-regulated lncRNA or mRNA. The lines between lncRNAs and mRNAs represent the targeting relationships. [file Image_4.TIF]
